# Editorial: Healthy adipose tissue expansion

**DOI:** 10.3389/fcell.2023.1287533

**Published:** 2023-09-28

**Authors:** Kevin Y. Lee, Brice Emanuelli, Siegfried Ussar

**Affiliations:** ^1^ Department of Biomedical Sciences, Heritage College of Osteopathic Medicine, Ohio University, Athens, OH, United States; ^2^ The Diabetes Institute, Ohio University, Athens, OH, United States; ^3^ Novo Nordisk Foundation Center for Basic Metabolic Research, Faculty of Health and Medical Sciences, University of Copenhagen, Copenhagen, Denmark; ^4^ RG Adipocytes and Metabolism, Institute for Diabetes and Obesity, Helmholtz Diabetes Center, Helmholtz Zentrum München, German Research Center for Environmental Health GmbH, Neuherberg, Germany; ^5^ German Center for Diabetes Research (DZD), Neuherberg, Germany

**Keywords:** adipose tissue, metabolism, adipocyte, triglyceride (TAG), insulin

White adipose tissue expansion, resulting in body weight gain, is generally viewed unfavorably due to esthetic and health reasons. The latter results from the general understanding that excessive accumulation of fat in adipose tissues results in impaired adipose function predisposing the organism to the development of systemic insulin resistance, the metabolic syndrome, and a host of other comorbidities that lead to a significant rise in mortality risk and impose social and economic burdens on individuals and the society. Recent societal changes such as the body positivity movement, paired with research findings identifying a potentially healthy obese phenotype aimed to change our perception and definition of excessive body fat. However, various clinical studies have questioned the existence of a healthy obese phenotype, with most recent data suggesting that a metabolically healthy obesity is merely an intermediate state in the progression from weight gain to the development of insulin resistance and the metabolic syndrome (Bluher, 2020). To this end, most research articles aim to identify factors underlying the detrimental metabolic consequences of body weight gain focusing on the negative consequences of increased adiposity. However, adipose tissue, in addition to its important endocrine function, remains essential to maintain homeostasis during times of energy surplus and shortage, in ensuring an ample supply of energy. In addition, the role of adipose tissue regulating the immune system in the host defense against pathogens is also an emerging area of research (Barthelemy et al., 2023).

In contrast to other organs, adipose tissue is not a single discrete organ but organized in individual depots distributed throughout the body (Schoettl et al., 2018). Thus, the overarching theme of the articles of this Research Topic is to highlight that not all adipose tissue expansion is the same. It is especially important to consider the specialized white adipose tissue depots, such as in the bone marrow fat as summarized by Burkhardt et al. (*The benefits of adipocyte metabolism in bone health and regeneration*), the dermal adipose tissue (Zhang et al., 2015; Zhang et al., 2019), or epiploic fat surrounding the human colon (Krieg et al., 2022; Onogi and Ussar, 2022) that appear to function beyond energy storage, regulating host immune defense, and bone and stem cell function. Adipose depots expand in response to increased energy intake, but adipocyte differentiation, growth and turnover are also regulated by other local paracrine factors, such as those from the tissue resident immune cells (Altun et al., 2022). These processes are critical to maintain tissue homeostasis, the adipose tissue being arguably one of the most plastic tissues of the organism (Sakers et al., 2022).

In addition, there are tremendous differences in the metabolic consequences of adipose tissue expansion depending on the affected depot/anatomical location and its nature (hypertrophy vs. hyperplasia). This is important as the mode of fat mass gain and adipose tissue expansion differs greatly between individuals, influenced by ethnicity, gender and age. White (“*Adipose tissue expansion in obesity, health, and disease*”) provides an interesting summary of the current progress and challenges in assessing adipose tissue expansion in humans. Moreover, Li and Spalding (“*The regulation of adipocyte growth in white adipose tissue*”) discuss key molecular pathways necessary to facilitate adipocyte growth from lipid synthesis and turnover to the reactivation of the cell cycle program allowing ample cellular response to the massive size increase. These data are nicely complemented by a perspective from Meriin et al. (“*Egr1 plays a major role in the transcriptional response of white adipocytes to insulin and environmental cues*”), where the authors discuss the transcriptional effects of insulin action and its relation to adipocyte function and growth. This review also touches on the critical input of the circadian rhythm in regulating adipocyte function and expansion through dynamic regulation of lipolysis, thermogenesis, and creatine cycling (Ribas-Latre et al., 2021; Hepler et al., 2022).

However, studying the physiological consequences of expansion of individual adipose tissue depots is complicated by differences in function, mode of expansion and anatomical location between human and mice, which remain the most utilized experimental model system for metabolic research. These important differences are reviewed by Börgeson et al. (“*Of mice and men: Pinpointing species differences in adipose tissue biology*”). Adipose tissues are highly innervated and adipose tissue function, growth, and expansion are impacted by central nervous inputs. This and the reverse endocrine and nervous signaling of adipose tissue to the central nervous system are summarized in a review article by Puente-Ruiz and Jais (“*Reciprocal signaling between adipose tissue depots and the central nervous system*”). This review also highlights the complexity of this reciprocal signaling between the sympathetic nervous system and different cellular components of adipose tissue.

Overall, there is a clear consensus that adipose tissue and to some extent also adipose expansion is essential for a functional metabolism. Moreover, a picture is emerging indicating that continuous triglyceride turnover is a key element in maintaining adipocyte function and static storage of triglycerides associates with adipocyte dysfunction (Morigny et al., 2021). However, excessive and uncontrolled lipolysis can induce a local inflammatory response resulting in fibrosis that limits the ability of individual adipocytes to expand. This causes adipocyte death, further inflammation and spillover of lipids to other organs such as the skeletal muscle and the liver. Future studies, will need to harvest the insights from recent single cell sequencing approaches describing adipocyte and stromal heterogeneity to develop novel transgenic technologies to study adipocyte growth in a depot and single adipocyte, preadipocyte, or immune cell specific manner. We are convinced that retaining adipocyte function and its ability to expand will be promising approaches to retain or restore insulin sensitivity in the context of obesity, by maintaining some sort of healthy obesity, and potentially further enhancing the efficacy of currently available weight loss drugs targeting the central nervous system.
